# Characteristics of HIV-2 and HIV-1/HIV-2 Dually Seropositive Adults in West Africa Presenting for Care and Antiretroviral Therapy: The IeDEA-West Africa HIV-2 Cohort Study

**DOI:** 10.1371/journal.pone.0066135

**Published:** 2013-06-18

**Authors:** Didier K. Ekouevi, Eric Balestre, Patrick A. Coffie, Daouda Minta, Eugene Messou, Adrien Sawadogo, Albert Minga, Papa Salif Sow, Emmanuel Bissagnene, Serge P. Eholie, Geoffrey S. Gottlieb, François Dabis, IeDEA West Africa collaboration, Djimon Marcel Zannou, Carin Ahouada, Jocelyn Akakpo, Christelle Ahomadegbé, Jules Bashi, Alice Gougounon-Houéto, Angèle Azon-Kouanou, Fabien Houngbé, Sikiratou Koumakpaï, Florence Alihonou, Marcelline d’Almeida, Irvine Hodonou, Ghislaine Hounhoui, Gracien Sagbo, Leïla Tossa-Bagnan, Herman Adjide, Joseph Drabo, René Bognounou, Arnaud Dienderé, Eliezer Traore, Lassane Zoungrana, Béatrice Zerbo, Adrien Bruno Sawadogo, Jacques Zoungrana, Arsène Héma, Ibrahim Soré, Guillaume Bado, Achille Tapsoba, Diarra Yé, Fla Kouéta, Sylvie Ouedraogo, Rasmata Ouédraogo, William Hiembo, Mady Gansonré, Eugène Messou, Joachim Charles Gnokoro, Mamadou Koné, Guillaume Martial Kouakou, Clarisse Amani Bosse, Kouakou Brou, Achi Isidore Assi, Henri Chenal, Denise Hawerlander, Franck Soppi, Albert Minga, Yao Abo, Germain Bomisso, Serge Paul Eholié, Mensah Deborah Noelly Amego, Viviane Andavi, Zelica Diallo, Frédéric Ello, Aristophane Koffi Tanon, Serge Olivier Koule, Koffi Charles Anzan, Calixte Guehi, Edmond Addi Aka, Koffi Ladji Issouf, Jean-Claude Kouakou, Marie-Sylvie N’Gbeche, Pety Touré, Divine Avit-Edi, Kouadio Kouakou, Magloire Moh, Valérie Andoblé Yao, Madeleine Amorissani Folquet, Marie-Evelyne Dainguy, Cyrille Kouakou, Véronique Tanoh Méa-Assande, Gladys Oka-Berete, Nathalie Zobo, Patrick Acquah, Marie-Berthe Kokora, Tanoh François Eboua, Marguerite Timité-Konan, Lucrèce Diecket Ahoussou, Julie Kebé Assouan, Mabéa Flora Sami, Clémence Kouadio, Lorna Renner, Bamenla Goka, Jennifer Welbeck, Adziri Sackey, Seth Ntiri Owiafe, Christian Wejse, Zacarias José Da Silva, Joao Paulo, Amabelia Rodrigues, David da Silva, Candida Medina, Ines Oliviera-Souto, Lars Østergaard, Alex Laursen, Morten Sodemann, Peter Aaby, Anders Fomsgaard, Christian Erikstrup, Jesper Eugen-Olsen, Moussa Y Maïga, Fatoumata Fofana Diakité, Abdoulaye Kalle, Drissa Katile, Hamar Alassane Traore, Daouda Minta, Tidiani Cissé, Mamadou Dembelé, Mohammed Doumbia, Mahamadou Fomba, Assétou Soukho Kaya, Abdoulaye M Traoré, Hamady Traoré, Amadou Abathina Toure, Fatoumata Dicko, Mariam Sylla, Alima Berthé, Hadizatou Coulibaly Traoré, Anta Koïta, Niaboula Koné, Clémentine N'Diaye, Safiatou Touré Coulibaly, Mamadou Traoré, Naïchata Traoré, Man Charurat, Samuel Ajayi, Stephen Dapiap, Otu, Festus Igbinoba, Okwara Benson, Clément Adebamowo, Jesse James, Obaseki, Philip Osakede, John Olasode, Papa Salif Sow, Bernard Diop, Noël Magloire Manga, Judicael Malick Tine, Haby Signate Sy, Abou Ba, Aida Diagne, Hélène Dior, Malick Faye, Ramatoulaye Diagne Gueye, Aminata Diack Mbaye, Akessiwe Patassi, Awèrou Kotosso, Benjamin Goilibe Kariyare, Gafarou Gbadamassi, Agbo Komi, Kankoé Edem Mensah-Zukong, Pinuwe Pakpame, Annette Koko Lawson-Evi, Yawo Atakouma, Elom Takassi, Améyo Djeha, Ayoko Ephoévi-gah, Sherifa El-Hadj Djibril, François Dabis, Emmanuel Bissagnene, Elise Arrivé, Patrick Coffie, Didier Ekouevi, Antoine Jaquet, Valériane Leroy, Charlotte Lewden, Annie Sasco, Jean-Claude Azani, Gérard Allou, Eric Balestre, Franck Bohossou, Sophie Karcher, Jules Mahan Gonsan, Jérôme Le Carrou, Séverin Lenaud, Célestin Nchot, Karen Malateste, Amon Roseamonde Yao, Bertine Siloué, Gwenaelle Clouet, Hugues Djetouan, Alexandra Doring, Adrienne Kouakou, Elodie Rabourdin, Jean Rivenc, Xavier Anglaret, Boubacar Ba, Jean Bosco Essanin, Andrea Ciaranello, Sébastien Datté, Sophie Desmonde, Jean-Serge Elvis Diby, Geoffrey S. Gottlieb, Apollinaire Gninlgninrin Horo, Serge N'zoré Kangah, Denis Malvy, David Meless, Aida Mounkaila-Harouna, Camille Ndondoki, Caroline Shiboski, Rodolphe Thiébaut, PAC-CI, Abidjan

**Affiliations:** Dept of Infectious Diseases, Aarhus University Hospital; Dept of Infectious Diseases, Aarhus University Hospital; Dept of Infectious Diseases, Odense University Hospital; Bandim Health Project; Dept. of Virology, Statens Serum Institut, Copenhagen; Dept. of Clinical Immunology; Dept. of Infectious Diseases, Hvidovre Hospital, Copenhagen; UATH, ***Abuja***; UBTH, ***Benin*** ** ***City***; OATH, ***Ile-Ife***; Principal Investigator, Bordeaux, France; Co-Principal Investigator, Abidjan, Côte d’Ivoire; Bordeaux, France; Abidjan, Côte d’Ivoire; Abidjan, Côte d’Ivoire; Bordeaux, France; Bordeaux, France; Bordeaux, France; Bordeaux, France; Abidjan, Côte d’Ivoire; Abidjan, Côte d’Ivoire; Bordeaux, France; Abidjan, Côte d’Ivoire; Bordeaux, France; Abidjan, Côte d’Ivoire; Bordeaux, France; Abidjan, Côte d’Ivoire; Abidjan, Côte d’Ivoire; Bordeaux, France; Abidjan, Côte d’Ivoire; Abidjan, Côte d’Ivoire; Bordeaux, France; Abidjan, Côte d’Ivoire; Bordeaux, France; Abidjan, Côte d’Ivoire; Bordeaux, France; Pessac, France; Bordeaux, France; Bamako, Mali; Abidjan; Boston, USA; Abidjan, Côte d’Ivoire; Bordeaux, France; Abidjan, Côte d’Ivoire; Seattle, USA; Abidjan, Côte d’Ivoire; Abidjan, Côte d’Ivoire; Bordeaux, France; Abidjan, Côte d’Ivoire; Bordeaux, France; Bordeaux, France; San Francisco USA; Bordeaux, France; 1 Université Bordeaux Segalen, ISPED, Centre INSERM U897- Epidémiologie-Biostatistique, Bordeaux, France; 2 Programme PACCI, Abidjan, Côte d’Ivoire; 3 Département des Sciences Fondamentales et Santé Publique, Université de Lomé, Lomé, Togo; 4 Département de Dermatologie et Infectiologie, UFR Sciences Médicales, Université Félix-Houphouët Boigny, Abidjan, Côte d’Ivoire; 5 Centre de Prise en Charge des Personnes vivant avec le VIH, Hôpital du Point G, Bamako, Mali; 6 ACONDA-CePReF Adultes, Abidjan, Côte d’Ivoire; 7 Hôpital de jour, CHU Souro Sanou, Bobo Dioulasso, Burkina-Faso; 8 Centre Médical de Suivi de Donneurs de Sang, Projet PRIMO-CI, Abidjan, Côte d’Ivoire; 9 Service des Maladies Infectieuses et Tropicales, CHU de Fann, Dakar, Sénégal; 10 Service des Maladies Infectieuses et Tropicales, CHU de Treichville, Abidjan, Côte d’Ivoire; 11 Departments of Medicine and Global Health, University of Washington, Seattle, Washington, United States of America; University of Pittsburgh, United States of America

## Abstract

**Background:**

HIV-2 is endemic in West Africa. There is a lack of evidence-based guidelines on the diagnosis, management and antiretroviral therapy (ART) for HIV-2 or HIV-1/HIV-2 dual infections. Because of these issues, we designed a West African collaborative cohort for HIV-2 infection within the framework of the International epidemiological Databases to Evaluate AIDS (IeDEA).

**Methods:**

We collected data on all HIV-2 and HIV-1/HIV-2 dually seropositive patients (both ARV-naive and starting ART) and followed-up in clinical centres in the IeDEA-WA network including a total of 13 clinics in five countries: Benin, Burkina-Faso Côte d’Ivoire, Mali, and Senegal, in the West Africa region.

**Results:**

Data was merged for 1,754 patients (56% female), including 1,021 HIV-2 infected patients (551 on ART) and 733 dually seropositive for both HIV-1 and HIV 2 (463 on ART). At ART initiation, the median age of HIV-2 patients was 45.3 years, IQR: (38.3–51.7) and 42.4 years, IQR (37.0–47.3) for dually seropositive patients (p = 0.048). Overall, 16.7% of HIV-2 patients on ART had an advanced clinical stage (WHO IV or CDC-C). The median CD4 count at the ART initiation is 166 cells/mm^3^, IQR (83–247) among HIV-2 infected patients and 146 cells/mm^3^, IQR (55–249) among dually seropositive patients. Overall, in ART-treated patients, the CD4 count increased 126 cells/mm^3^ after 24 months on ART for HIV-2 patients and 169 cells/mm^3^ for dually seropositive patients. Of 551 HIV-2 patients on ART, 5.8% died and 10.2% were lost to follow-up during the median time on ART of 2.4 years, IQR (0.7–4.3).

**Conclusions:**

This large multi-country study of HIV-2 and HIV-1/HIV-2 dual infection in West Africa suggests that routine clinical care is less than optimal and that management and treatment of HIV-2 could be further informed by ongoing studies and randomized clinical trials in this population.

## Introduction

Human Immunodeficiency Virus type 2 (HIV-2) was first isolated in 1986 [1]–[3]. As compared to the global HIV-1 pandemic, the HIV-2 epidemic has remained essentially confined to West Africa with a limited spread to other regions [4]. In West Africa, between 10–20% of HIV infections are HIV-2 or HIV-1/HIV-2 dual seropositive [5], [6] corresponding to 1–2 million people living with this virus in the region [7] although prevalence has been waning over the last few decades [6].

Compared with HIV-1 infection, HIV-2 infection is characterized by a much longer asymptomatic stage, lower plasma viral load, slower CD4 cell count decline, lower AIDS-related mortality rate [4], [8]–[11], lower rates of mother-to-child transmission [12], [13], [14], genital tract shedding [15] and sexual transmission [16], [17]. Nonetheless, a significant proportion of HIV-2 infected individual’s progress to AIDS and may benefit from antiretroviral therapy (ART) [18].

All antiretrovirals to date have been developed to inhibit HIV-1 replication and many of them are not active against HIV-2. HIV-2 is intrinsically resistant to the non-nucleoside reverse transcriptase inhibitors (NNRTIs) and the fusion inhibitor, enfurvirtide [19]–[21]. Moreover, only three protease inhibitors (PI) have potent activity against HIV-2: lopinavir, saquinavir and darunavir [21], [22]. The nucleoside/tide reverse transcriptase inhibitors (NRTIs) appear to be equally potent against HIV-2 as for HIV-1, however different resistance pathways and a lower genetic barrier to resistance make their use problematic [23], [24]. The newer antiretroviral classes, the integrase inhibitors (INI) show promising in vitro activity against HIV-2 isolates [25]–[28]. Preliminary studies among HIV-2 infected patients showed that the INI class drugs (raltegravir and elvitegravir) is an interesting option among HIV-2 naïve patients as well as those who experience therapeutic failure [29], but remains to be formally evaluated through clinical trials. Of note, INI are already included in some guidelines for HIV-2 infection [22].

There is a lack of evidence-based guidelines on the diagnosis, management and ART use for HIV-2 or HIV-1/HIV-2 dual infections [21], [22], [30], [31] especially in resource-limited settings [21]. The main reasons are that first the efficacy of ART in HIV-2 infection has not been evaluated through randomized clinical trials [7], [32] and second observational cohorts generally have had small numbers of patients and shown relatively poor outcome [33]–[38] although emerging data suggest LPV/r-based regimens have reasonable efficacy [35].

Because of these issues, we designed and organized a West African collaborative cohort of HIV-2 infection within the framework of the International epidemiological Database to evaluate AIDS (IeDEA) [39]. We collected data on all HIV-2 and HIV-1/HIV-2 dually seropositive patients both ARV-naïve and ARV-treated and followed-up in clinical centres in the IeDEA-WA network in the West Africa region. Here, we report the characteristics of HIV-2 and HIV-1/HIV-2 dually seropositive adults in West Africa presenting for care and on ART.

## Methods

### Description of the Cohort

**Figure 1:**
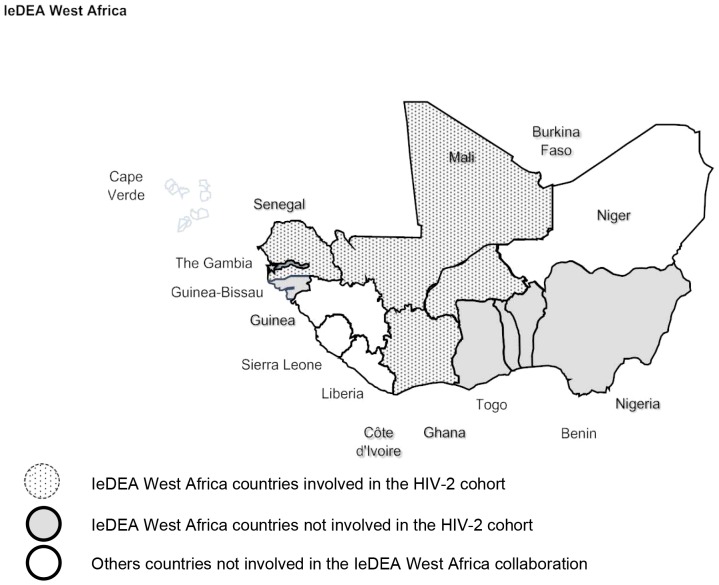
Geographical location of the clinical centres participating to the IeDEA-WA-HIV-2 cohort.

The organization and structure of the Sub-Saharan IeDEA cohorts have been previously described [39] and the HIV-2 cohort collaboration is a newly formed network of HIV-2 and dually HIV-1/HIV-2 seropositive patients in West Africa embedded in the IeDEA-West Africa group. A total of 13 clinics (all are located in urban areas), in five countries (Benin, Burkina-Faso, Cote d’Ivoire, Mali, Senegal) are participating (Figure 1 and Table 1). All of the sites have the capacity to measure CD4 cell counts, hematology and chemistries. Only two countries (Senegal and Cote d’Ivoire) had an equipped laboratory for performing HIV-2 plasma viral load measurements [40]–[41], however they were not routinely performed.

**Table 1:** Enrollment per clinical center and per country. IeDEA-WA-HIV-2 cohort, as of December 2011.

	HIV-2-infected patients (%)		Dually seropositive patients (%)		Total
	ARV-naive	ART	ARV-naive	ART	
**Benin**					
Service de Médecine Interne, CNHU, Cotonou	7 (1.5)	4 (0.7)	6 (2.2)	9 (1.9)	26
**Burkina-Faso**					
CHU Yalgado, Ouagadougou	44 (9.4)	54 (9.8)	35 (13.0)	66 (14.3)	199
Hôpital de jour, Bobo-Dioulasso	38 (8.1)	41 (7.4)	1 (0.4)	1 (0.2)	81
**Côte d’Ivoire, Abidjan**					
SMIT, CHU Treichville	175 (37.2)	112 (20.3)	100 (37.1)	90 (19.4)	477
CNTS, Treichville	85 (18.1)	33 (6.0)	18 (6.7)	36 (7.8)	172
CIRBA, Treichville	48 (10.2)	46 (8.4)	21 (7.8)	31 (6.7)	146
USAC, Treichville	9 (1.9)	37 (6.7)	2 (0.7)	15 (3.2)	63
MTCT-Plus, Yopougon	11 (2.3)	1 (0.2)	2 (0.7)	6 (1.3)	20
CePReF, Yopougon	44 (9.4)	113 (20.5)	77 (28.5)	179 (38.7)	413
**Mali, Bamako**					
Hôpital point G	1 (0.2)	17 (3.1)	2 (0.7)	5 (1.1)	25
CHUGabriel Touré	8 (1.7)	19 (3.5)	6 (2.2)	25 (5.4)	58
**Senegal, Dakar**					
SMIT, CHU Fann	–	74 (13.4)	–	–	74
TOTAL	470(100.0)	551(100.0)	270 (100.0)	463(100.0)	1754(100.0)

### Schedule of Follow-up

ART was provided by the individual national treatment programs according to their individual treatment algorithms. After initiation into care, patients were typically followed every six months, or were seen in between visits for any intercurrent illness. CD4 count were measured every six months.

### Data Collection

Standardized questionnaires capturing the relevant information on HIV-2 care have been developed with an electronic database implemented at the site level. All sites completed retrospectively and then prospectively the specific questionnaires and have entered the data in the unique IeDEA-WA HIV-2 database. The databases from each site are sent every six months to the Regional Centre in Abidjan, Côte d’Ivoire, and Bordeaux, France using compression/encryption software. Data collected include: 1) Baseline demographics: birth date, gender, HIV clinical stage (WHO or CDC stage), ART initiated, clinical assessment, medical history, 2) Follow-up: Clinical assessment (tuberculosis, other diseases/infection, HIV clinical stage, weight, height, medications such as antiretroviral drugs and cotrimoxazole), 3) Biological data: including CD4, haemoglobin, ALAT, ASAT, plasma HIV RNA viral load (when available, and 4) Outcomes: death, loss to follow-up, and transferred out.

### Data Management

Clinical and biological questionnaires are collected and centralized at the IeDEA West Africa, Regional office in the Programme PACCI in Abidjan where they are checked for accuracy and completeness and transfer to the INSERM U897 Epidemiology centre in Bordeaux, France, for statistical analysis and cohort description. The database used for the current manuscript included information recorded at enrolment as well as during follow-up up to December 2011.

### Statistical Analysis

Continuous variables are described by their median value and interquartile range (IQR); categorical variables are described as percentages. All statistical tests are two-sided, with a type I error of 5%.

### Ethical Aspects

The study was designed and performed in accordance with the Declaration of Helsinki and was approved by the National ethics committee (IRB) from Benin: Comité National d'Ethique pour la Recherche en Santé, from Burkina-Faso: Comité d'Ethique pour la Recherche en Santé, from Côte d’Ivoire: Comité National d'Ethique et de la Recherche, from Mali: Comité National d’Ethique pour la Santé et les Sciences de la vie and from Senegal: Comité National d'Ethique pour la Recherche en Santé. A waiver of informed consent was granted by the Institutional Review Boards because we used data collected in routine and used by the national program for monitoring HIV programs. The study procedure did not involve any personal contact with the patients.

## Results

As of December 2011 the cohort has captured baseline and follow-up data from 1,754 (56% female) HIV-2 or dually seropositive patients, of whom 1,014 (57.8%) have initiated ART (Table 1). Overall, 1,021 HIV-2 infected patients (551 on ART) and 733 dually seropositive for HIV-1 and HIV 2 (463 on ART) have been enrolled (Table 2).

**Table 2:** Socio-demographic, clinical, biological and therapeutic characteristics of HIV-2 and dual seropositive patients. IeDEA-WA-HIV-2 cohort.

	HIV-2-infected patients (N = 1021)		Dually seropositive patient (N = 733)		p^1^	p^2^
	ARV-naïve (n = 470)	ART (n = 551)	ARV-naïve (n = 270)	On ART (n = 463)		
**Age (years)**						
Median (IQR)	42.8 (34.9–49.4)	45.3 (38.3–51.7)	40.6 (34.4–47.2)	42.4 (37.0–47.3)	<0.001	0.048
<40	163 (34.8)	144 (26.1)	117 (43.3)	161 (34.8)	<0.001	0.061
40–49	175 (37.3)	185 (33.6)	87 (32.2)	196 (42.3)		
> = 50	100 (21.3)	143 (26.0)	44 (16.3)	63 (13.6)		
Unknown	31 (6.6)	79 (14.3)	22 (8.2)	43 (9.3)		
**Gender**					0.060	0.732
Male	189 (40.2)	234 (42.5)	104 (38.5)	224 (48.4)		
Female	279 (59.4)	313 (56.8)	162 (60.0)	236 (51.0)		
Unknown	2 (0.4)	4 (0.7)	4 (1.5)	3 (0.6)		
**WHO/CDC stage**					0.001	<0.001
WHO I/II or CDC-A	118 (25.1)	78 (14.2)	29 (10.7)	45 (9.7)		
WHO III or CDC-B	45 (9.6)	246 (44.6)	57 (21.1)	254 (54.9)		
WHO IV or CDC-C	9 (1.9)	92 (16.7)	7 (2.6)	83 (17.9)		
Unknown	298 (63.4)	135 (24.5)	177 (65.6)	81 (17.5)		
**CD4 count**						
Median (IQR)	535(319–820)	166(83–247)	282(108–518)	146(55–249)	0.044	<0.001
<200	63 (13.4)	265 (48.1)	82 (30.4)	235 (50.8)	0.100	<0.001
200–349	63 (13.4)	121 (22.0)	46 (17.0)	125 (27.0)		
350–499	82 (17.5)	25 (4.5)	40 (14.8)	15 (3.2)		
500–799	114 (24.2)	13 (2.4)	41 (15.2)	11 (2.4)		
>800	117 (24.9)	3 (0.5)	19 (7.0)	2 (0.4)		
Unknown	31 (6.6)	124 (22.5)	42 (15.6)	75 (16.2)		
**ART**						
Not on ART	470	–	270	–	<0.001	
NRTIs+PI	–	463 (84.0)	–	305 (65.9)		
NRTIs+NNRTI	–	39 (7.1)	–	123 (26.6)		
3 NRTIs	–	48 (8.7)	–	29 (6.3)		
Mono/bi-therapy	–	1 (0.2)	–	6 (1.2)		
**Year of enrolment^$^**						
1992–2003	62 (13.5)	51 (9.3)	88 (33.0)	81 (17.5)	<0.001	<0.001
2004–2005	121 (26.4)	126 (22.9)	89 (33.3)	149 (32.2)		
2006–2007	96 (21.0)	155 (28.1)	28 (10.5)	100 (21.6)		
2008–2011	179 (39.1)	219 (39.7)	62 (23.2)	133 (28.7)		

IQR: Interquartile range, ART: antiretroviral treatment.

p^1^: comparison between two group of patient on ART, p^2^ =  comparison between the two groups of patients in care.

PI: protease inhibitors, NRTI: nucleoside reverse transcriptase inhibitors, NNRTI: non nucleoside reverse transcriptase inhibitors.

*$: 15 missing data for enrolment date in care.*

Among patients whom were ART-naive, the median age was 42.8 years, interquartile range (IQR) (34.9–49.4) for HIV-2 patients and 40.6 years, IQR (34.4–47.2) for dually seropositive patients (p<0.001). At enrolment, 49.1% of HIV-2 patients had CD4 count ≥500 cells/mm^3^ versus 22.2% for dually seropositive patients (p<0.001) (Table 2).

At ART initiation, the median age of HIV-2 patients was 45.3 years, IQR (38.3–51.7) and 42.4 years, IQR (37.0–47.3) for dually seropositive patients (p = 0.048). In addition, the proportion of patients aged ≥50 years was twice as high in HIV-2 patients compared with dually seropositive patients (26.0% vs 13.6%, p<0.001). Of note, 16.7% of HIV-2 patients had an advanced clinical stage (WHO stage IV or CDC-C), a figure comparable to the 17.9% observed among dually seropositive patients.

In all clinical centers, the most commonly used first-line ART regimen was a protease inhibitors (PI) based regimen, which was initiated in 84.0% of HIV-2 patients and 65.9% for dually seropositive patients. The most common PI based regimen prescribed was lopinavir-ritonavir (Aluvia® or Kaletra®) for 36.4% HIV-2 patients and 26.9% for dually seropositive patients on ART. Despite the fact that NNRTIs are not recommended among HIV-2 patients due to intrinsic resistance, it was initiated in 39 (7.1%) of HIV-2 patients and 123 (26.6%) of dually seropositive patients (Table 2). Finally, 3 NRTIs regimens were prescribed for 8.7% of HIV-2 infected patients and for 6.3% of dually seropositive patients. The most prescribed NRTI regimens among HIV-2 infected patients were the followings zidovune+lamivudine (3TC) (63.3%), stavudine (d4T) +3TC (30.5%), tenofovir+(emtricitabine or 3TC) (2.7%) and didanosine+ (abacavir+d4T) (1.1%).

**Figure 2:**
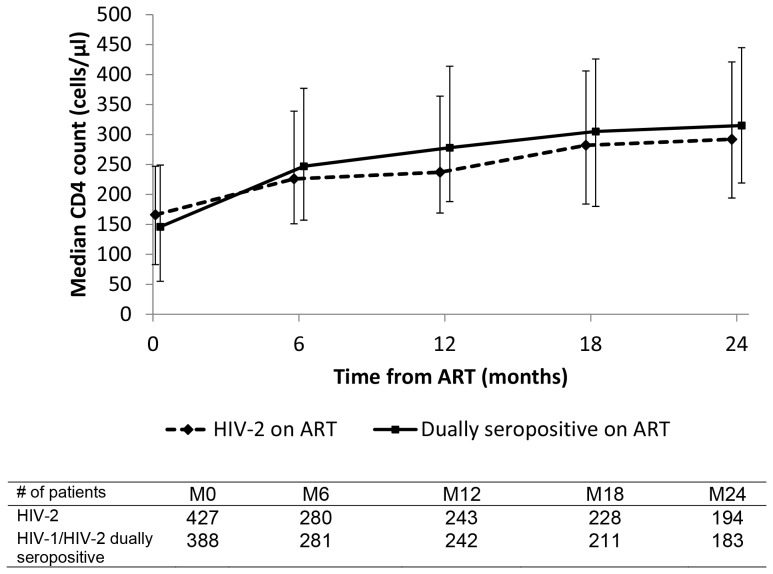
Median CD4 count (cells/µl) and interquartile range, from ART initiation in the HIV-2 cohort, IeDEA West Africa collaboration.

The median CD4 count at the ART initiation is 166 cells/mm^3^, IQR (83–247) among HIV-2 infected patients and 146 cells/mm^3^, IQR (55–249) among dually seropositive patients (p<0.001). At 24 months on ART, the median CD4 count increased 126 cells/mm^3^ for HIV-2 patients and 169 cells/mm^3^ for dually seropositive patients (Figure 2). Of 551 HIV-2 patients on ART, 32 (5.8%) died and 56 (10.2%) were considered lost to follow-up during the median time on ART of 2.4 years, IQR (0.7–4.3). Of 463 dually seropositive patients on ART, 21 (4.6%) died and 46 (9.9%) were considered lost to follow-up during a median follow on ART of 2.6 years (0.9–5.5). Table 3 summarizes the follow-up characteristics of both ARV-naïve and ARV-treated HIV-2 and dually seropositive patients.

**Table 3:** Follow-up characteristics. IeDEA-WA-HIV-2 cohort.

	HIV-2-infected patients		Dually seropositive patient	
	ARV-naïve (n = 470)	ART (n = 551)	ARV-naïve (n = 270)	ART (n = 463)
**Duration**				
Median in years (IQR)	1.1 (0–3.8)	2.4 (0.7–4.3)	0.1 (0–2.0)	2.6 (0.9–5.5)
<12 months (%)$	229 (48.7)	161 (29.2)	183 (67.8)	126 (27.2)
12–23 months (%)	60 (12.8)	87 (15.8)	19 (7.0)	73 (15.8)
24–37 months (%)	40 (8.5)	72 (13.1)	19 (7.0)	45 (9.7)
>37 months (%)	141 (30.0)	231 (41.9)	49 (18.2)	219 (47.3)
**Status (%)**	n (%)	n (%)	n (%)	n (%)
Alive	355 (75.5)	449 (81.5)	190 (70.4)	387 (83.6)
Deceased	7 (1.5)	32 (5.8)	10 (3.7)	21 (4.6)
LTFU*	96 (20.4)	56 (10.2)	69 (25.6)	46 (9.9)
Dropped out	5 (1.1)	4 (0.7)	0(0.0)	1 (0.2)
Transferred -out	7 (1.5)	10 (1.8)	1 (0.3)	8 (1.7)

IQR: Interquartile range, ART: antiretroviral treatment initiated.

* Number of patients lost to follow-up reported and recorded in the HIV-2 database.

$ patients without any follow-up are included.

## Discussion

The IeDEA-WA-HIV-2 cohort is a multi-centre, multi-country collaboration on HIV-2 and dually seropositive patients in West Africa, the only region of the world where the two viruses are endemic (HIV-2) and epidemic (HIV-1). This is one of the largest datasets in the world on HIV-2 and provides the opportunity to study HIV-2 in resource-limited settings (RLS). The only other large HIV-2 database is the ACHIEV_2_E HIV-2 Collaboration in Europe and North America [35]. The IeDEA-WA HIV-2 cohort aims, first, to build and strengthen an operational and clinical research network that describes HIV-2 case management in RLS. Treatment outcomes and their determinants are being investigated by pooling individual patient data, and should thus appropriately inform policies and programmes by examining various models of care, clinical and operational outcomes. The network is reflecting on a similar successful experience of adult treatment centres in lower income countries [39] and aims to rapidly bridge the gap in clinical and programmatic information for people living with HIV-2. Furthermore, this collaboration includes not only patients on ART but also patient “in care” but ARV-naive. In addition, we have also implemented an HIV-2 and HIV-1/HIV-2 Drug Resistance Database that will allow us to conduct surveys of genotypic resistance and virological response in the future. Moreover, we are in the process of implementing and standardizing plasma HIV-2 RNA viral load quantification across sites.

It is important to note that several centres identified as possible sites of enrolment and follow-up of HIV-2 patients in the West African region did not participate in this collaboration because they do not perform routinely HIV serologic testing that discriminates HIV-1 from HIV-2, (e.g. Nigeria, which has the largest number of HIV-infected patients in West Africa).

In West Africa, rapid HIV assays are often used for the diagnosis of HIV-1 or HIV-2 or dual HIV-1/HIV-2 infection. This strategy is based on the demonstration of virus-specifics antibodies using enzyme-linked immunosorbent assay based-technique [21], [30], [42], [43]. In the West African region, current serological tests for the diagnosis of HIV-2 include: 1) For screening purposes: Determine, ELISA and Murex ICEVIH1.0.2., 2) for confirmatory testing and HIV-1, HIV-2 differentiation: Genie II HIV-1/2 (Bio-Rad, Marnes la Coquette, France), Immunocomb HIV 1&2 (Orgenics Ltd, Yavne, Israel), SD Bioline (Standard Diagnostics, Inc., Korea) or HIV-2 and HIV-1 Western blots. 3) Final confirmation is made when available with Pepti-LAV (Bio-Rad), Western Blot, HIV DNA PCR (in house), and/or Inno-LIA HIV I/II (Innogenetics, Belgium) depending on the laboratories. A specific survey on serological testing is ongoing within this collaboration in order to propose soon a common diagnosis algorithm for all the participating centers.

HIV-2 may be underreported because antibody cross-reactivity between HIV-1 and HIV-2 is common and frequently results in misdiagnosis of HIV-2 as HIV-1 or dual infection [44], [45]. Therefore screening tests need high sensitivity for HIV-2, while confirmatory testing may require multiple steps in order to reliably distinguish between HIV-1, HIV-2, and HIV-1/HIV-2 dual infection. The consequence of misdiagnosis that results in HIV-2 and dually seropositive patients going on treatment that ignores their HIV-2 as reported here with 7.1% of HIV-2 patients who had initiated NNRTI regimens because the clinician did not know they had HIV-2 at the time of ART initiation. Dual infection can be proven only by the presence of both HIV-1 and HIV-2 DNA or RNA by specific PCR the isolation of both viruses from the same individual [46]. However plasma HIV-2 RNA may be undetectable using current assays, it cannot be used as a diagnostic test. HIV-2 proviral DNA may be low or repeatedly negative in some asymptomatic individuals, making confirmation of diagnosis difficult [47].

CD4 count is the most readily available means for monitoring disease progression in HIV-2-infected patients. However, CD4 count often will not increase as rapidly as it generally occurs with successful therapy of HIV-1 mono-infection [33], [34], [48]. It has been reported that there is less and slower CD4 count recovery in older patients with HIV-1, limiting the CD4 interpretation of CD4 trajectory in older HIV-2 patients on ART [49]. Thus, there is no validated definition of immunological failure in HIV-2 infection. Consequently, in West Africa, it is difficult to manage HIV-2 patients with ART failure since there are no commercially available viral load and resistance tests for HIV-2 infection [50]–[52], There are indeed very limited 1^st^ and 2^nd^ line treatment options for HIV-2 in most RLS in West Africa. The lack of available second-line HIV-2 therapy options should be considered when choosing first-line ART, as the initial regimen choice, narrows later treatment options. Additionally, in HIV-1/HIV-2 dual infection, clinical management needs to focus on controlling both viruses with agents that are active against both HIV-1 and HIV-2 [21].

Finally our large study of HIV-2 and HIV-1/HIV-2 dual infection in West Africa suggests that routine clinical care is less than optimal and that management and treatment of HIV-2 could be further informed by ongoing studies and randomized clinical trials in this population.
